# A Four‐Gene Autophagy‐Related Prognostic Model Signature and Its Association With Immune Phenotype in Lung Squamous Cell Carcinoma

**DOI:** 10.1002/cnr2.70000

**Published:** 2024-10-23

**Authors:** Lumeng Luo, Jiaying Deng, Qiu Tang

**Affiliations:** ^1^ Department of Radiation Oncology Women's Hospital, School of Medicine, Zhejiang University Zhejiang China; ^2^ Zhejiang Provincial Key Laboratory of Precision Diagnosis and Therapy for Major Gynecological Diseases, Women's Hospital, Zhejiang University School of Medicine Hangzhou People's Republic of China; ^3^ Zhejiang Provincial Clinical Research Center for Obstetrics and Gynecology Zhejiang China; ^4^ Department of Radiation Oncology Fudan University Shanghai Cancer Center Shanghai China; ^5^ Department of Oncology Shanghai Medical College, Fudan University Shanghai China

**Keywords:** autophagy, bioinformatic analysis, immune infiltration, immune landscape, lung squamous cell carcinoma, prognostic signature

## Abstract

**Background:**

In the era of immunotherapy, there is a critical need for effective biomarkers to improve outcome prediction and guide treatment decisions for patients with lung squamous cell carcinoma (LUSC). We hypothesized that the immune contexture of LUSC may be influenced by tumor intrinsic events, such as autophagy.

**Aims:**

We aimed to develop an autophagy‐related risk signature and assess its predictive value for immune phenotype.

**Methods and Results:**

Expression profiles of autophagy‐related genes (ARGs) in LUSC samples were obtained from the TCGA and GEO databases. Survival analyses were conducted to identify survival‐related ARGs and construct a risk signature using the Random Forest algorithm. Four ARGs (CFLAR, RGS19, PINK1, and CTSD) with the most significant prognostic value were selected to construct the risk signature. Patients in the high‐risk group exhibited worse prognosis than those in the low‐risk group (*p* < 0.0001 in TCGA; *p* < 0.01 in GEO) and the risk score was identified as an independent prognostic factor. We observed that the high‐risk group displayed an immune‐suppressive status and showed higher levels of infiltrating regulatory T cells and macrophages, which are associated with poorer outcomes. Additionally, the risk score exhibited a significantly positive correlation with the expression of PD‐1 and CTLA4, as well as the estimate score and immune score.

**Conclusion:**

This study provided an effective autophagy‐related prognostic signature, which could also predict the immune phenotype.

**Abbreviations**
ARG: autophagy‐related gene
AUC: area under the curve
DEGs: differentially expressed genes
ESTIMATE: Estimation of Stromal and Immune cells in Malignant Tumor
GEO: Gene Expression Omnibus
GSEA: gene set enrichment analysis
ICIs: immune checkpoint inhibitors
KEGG: Kyoto Encyclopedia of Genes and Genomes
LUSC: lung squamous cell carcinoma
NSCLC: non‐small cell lung cancer
OS: overall survival
PD‐1: programmed cell death protein‐1
PD‐L1: programmed death ligand‐1
ROC: receiver operating characteristic
ssGSEA: Single‐Sample Gene Set Enrichment Analysis
TCGA: The Cancer Genome Atlas

## 1 Introduction

Lung cancer is the second most common cancer, and is the leading cause of cancer death worldwide [1]. Lung squamous cell carcinoma (LUSC), a subtype of non‐small cell lung cancer (NSCLC), is one of the main histological types of lung cancer [2]. Unlike lung adenocarcinoma, no first‐line targeted therapies are clinically available in LUSC patients [3]. In recent years, immune checkpoint inhibitors (ICIs) targeting the programmed death ligand‐1 (PD‐L1)/PD‐1 immune checkpoint axis have shown promising results in the treatment of NSCLC, including LUSC [4]. However, only a fraction of patients seem to benefit from immunotherapy, likely due to tumor intrinsic heterogeneity and variation in the tumor immune microenvironment (TIME) [4]. Given that cancer cells rely on remodeling their microenvironment and evading the tumor immune microenvironment, it is crucial to reprogram killer cells to overweigh the hypo‐function induced by cancer cells, induced by cancer cells, particularly concerning the role of autophagy across various cancer types [5]. Besides, more effective biomarkers for prognosis and optimal treatment selection are urgently needed. Finding more effective and precise methods for predicting outcomes in LUSC is vital.

Autophagy, an intracellular lysosomal degradation pathway that supports nutrient recycling and metabolic adaptation, is supposed to protect cells and tissues from stressors in normal physiological processes [6]. There is growing evidence suggesting that autophagy also plays an important role in various pathological processes, especially in cancer [6]. In the earliest stages of tumorigenesis, autophagy may limit the development of tumors [7]; however, during the advanced stages of tumors, autophagy is upregulated and promotes tumor cell proliferation by absorbing nutrients and energy derived from degraded proteins and organelles [8]. Autophagy can help cells cope with intracellular and environmental stresses, such as hypoxia, nutrient shortage, or cancer therapy, thereby favoring tumor progression [6, 9, 10]. In the tumor microenvironment, autophagy serves as an important regulator of immune responses, modulating the functions of immune cells and the production of cytokines [11]. Autophagy appears to be a “double‐edged sword” in immune cells within the tumor microenvironment, as it can either promote or suppress tumor development depending on the properties of the tumor and cell types involved.

With the remarkable success of PD‐L1/PD‐1 inhibitors and other ICIs in NSCLC, the question arises regarding the impact of autophagy on cancer treatments in the era of immunotherapy. Recent studies have unveiled a connection between anticancer immunity and various cell death mechanisms, particularly autophagy [12]. In tumors resistant to ICIs, the simultaneous activation of pyroptosis, ferroptosis, and necroptosis alongside ICIs has led to a synergistic enhancement in anticancer efficacy [13, 14]. Hence, a thorough comprehension of autophagy‐related genes (ARGs) associated with the immune microenvironment would facilitate the expansion of immunotherapies and the exploration of predictive biomarkers for designing patient‐tailored combination treatments. Currently, few studies have systematically investigated the correlation between autophagy and the immune microenvironment of LUSC. Furthermore, whether ARGs and immune infiltration levels could serve as prognostic factors in LUSC subtypes remains to be fully elucidated.

In this study, expression profiles of autophagy‐related genes (ARGs) in LUSC samples were analyzed from TCGA and GEO. We developed a prognostic model signature based on four ARGs with the most significant prognostic value by random forest algorithm. Patients were divided into high and low‐risk groups. Gene set enrichment analysis (GSEA) and correlation analysis with immune cells and checkpoints were performed. The results shine light on clarifying the association of ARGs and immune escape, and establish a more personalized precision predicting model for immunotherapy.

## 2 Materials and Methods

### 2.1 Data Collection

We collected all transcriptome profiles of LUSC available in the TCGA database as of January 17, 2023 (https://portal.gdc.cancer.gov/) [15]. Corresponding clinical information of these patients including gender, age, pathological stage, TNM stage, follow‐up time, survival status was also obtained from TCGA (Samples missing any clinical characteristics were excluded and samples of which OS ≤ 30 days were also excluded because these patients probably died of unpredictable factors). Our study included the expression profile of 32 normal samples and 326 LUSC samples. As the status of distant metastasis is missing in lots of samples, we didn't take the status of M stage into consideration in the current study. Transcriptome data of GSE41271 from the GEO dataset (https://www.ncbi.nlm.nih.gov/geo/) were obtained, comprising 78 LUSC subtype samples with available clinical and survival data, utilized as an independent validation cohort.

### 2.2 Acquisition of ARGs

The Human Autophagy Database (HADb, http://www.autophagy.lu/) provides a complete and continuously updated list of human genes related to autophagy processes reported in PubMed or other common databases [16]. We obtained 232 ARGs from HADb, of which 224 genes were available in the expression profile from TCGA. The gene list is shown in Table S1.

### 2.3 Differentially Expressed Analysis of ARGs and Functional Enrichment Analysis

To compare the expression levels of ARGs between tumor and normal samples, a differential expression analysis of all ARGs was conducted based on the Wilcoxon test with the edgeR package under the R environment (version 3.6.3). The cutoff criterion for differentially expressed genes (DEGs) was set as *p* < 0.05 and |log2 fold change| > 1. The results were displayed using the pheatmap package. Subsequently, Kyoto Encyclopedia of Genes and Genomes (KEGG) pathway enrichment analysis was performed to explore the main roles of dysregulated differentially expressed ARGs (DE‐ARGs) in LUSC. The cutoff criterion was set as *p* < 0.05 and Benjamin‐Hochberg adjusted *p* < 0.05. The analyses and visualization of results were conducted using the ClusterProfiler package in the R environment.

### 2.4 Survival‐Related ARGs (sARGs)

The ARGs associated with clinical outcomes in LUSC patients were identified as sARGs. sARGs were selected using the Kaplan–Meier method and verified using a log‐rank test conducted by the R packages survival and survminer. The cutoff value of the expression level of each gene was set at 50%, with *p* < 0.05. A total of 54 survival‐related ARGs were identified for subsequent research.

### 2.5 Construction and Validation of Autophagy‐Related Prognostic Signature

We used random survival forest (RSF) method for developing a prognosis model based on sARGs. The random forest algorithm is a machine learning strategy, which is based on the construction of many classification (decision) trees that are used to classify the input data vector [17]. Firstly, the RF algorithm used different bootstrapped and randomly split samples of the original dataset to build each decision tree. 30% of the samples were randomly split from the original dataset and set as cross‐validation set (test set), the rest 70% set as training set. Then a decision tree was constructed on each Bootstrap sample set. A variable importance (VIMP) measure can be calculated based on the impact that each variable effect on the out‐of‐bag prediction error as the respective variable values are randomly permuted. In this study, each sample has 54 features (sARGs). 10‐fold cross‐validation was used to select the features to be used to train random forest regression model. Through the calculation of variable importance scores of all features, we finally selected the top 4 sARGs with the highest variable importance scores to represent the training samples. At last, a random forest regression model was constructed using the training sample set consisting of the 4 sARGs by running randomForest package on R platform. The developed RSF prognosis model was then evaluated on the independent dataset (GEO dataset) where the RSF‐based score was derived for each sample. Then, to verify the validity and robustness of the RSF prognosis model, standard Kaplan–Meier survival curves were generated for different risk patient groups on the basis of the RSF‐based scores. Based on the optimal cutoff values obtained by the survminer R package, LUSC patients were classified as low‐risk and high‐risk according to their risk score. To appraise the prognostic performance of the model, Kaplan–Meier analysis and the log‐rank test were employed. Time‐dependent receiver operating characteristic (ROC) curves were depicted to evaluate the sensitivity and specificity using the timeROC R package [18]. Area under the curve (AUC) values were calculated from the ROC curves. We then performed univariate and multivariate Cox regression analyses to verify the prognostic value of the risk score. We took age, gender, TNM stage (M stage excluded) as candidate risk factors for regression analyses. We evaluated if all these factors are risk factors for poor prognosis by univariate Cox regression analysis, and then by multivariate Cox regression, further determined if the risk score could be utilized for predicting the prognosis of LUSC patients independently.

### 2.6 Gene Set Enrichment Analysis (GSEA)

GSEA was conducted to explore significant immune phenotypes between the high‐risk and low‐risk groups [19]. Gene sets involved in the negative‐regulation of immune response was imported from MSIGDB gmt file from Broad institute. GSEA was performed using Clusterprofiler R package and GSEABase R package. The cutoff criterion for statistically significant terms was set at *p* < 0.05.

### 2.7 Immune Cell Infiltration

Single‐Sample Gene Set Enrichment Analysis (ssGSEA) was used to quantify the relative infiltration of 28 immune cell types in the tumor microenvironment. GSVA R package was used in the analysis. Feature gene panels for each immune cell type were obtained from previous studies [20, 21]. The relative abundance of each immune cell type was represented by an enrichment score from ssGSEA analysis.

### 2.8 ESTIMATE Algorithm

The ESTIMATE (Estimation of Stromal and Immune cells in Malignant Tumor tissues using Expression data) algorithm was applied to the normalized expression matrix for estimating the stromal and immune scores using estimate R package (http://r‐forge.r‐project.org; repos = rforge, dependencies = TRUE) for each LUSC sample [22].

### 2.9 Statistical Analysis

Statistical analyses were conducted using the R software (version 3.6.3), with *p* < 0.05 considered significant.

## 3 Results

### 3.1 Identification of Differentially Expressed Autophagy‐Related Genes (ARGs)

**FIGURE 1:**
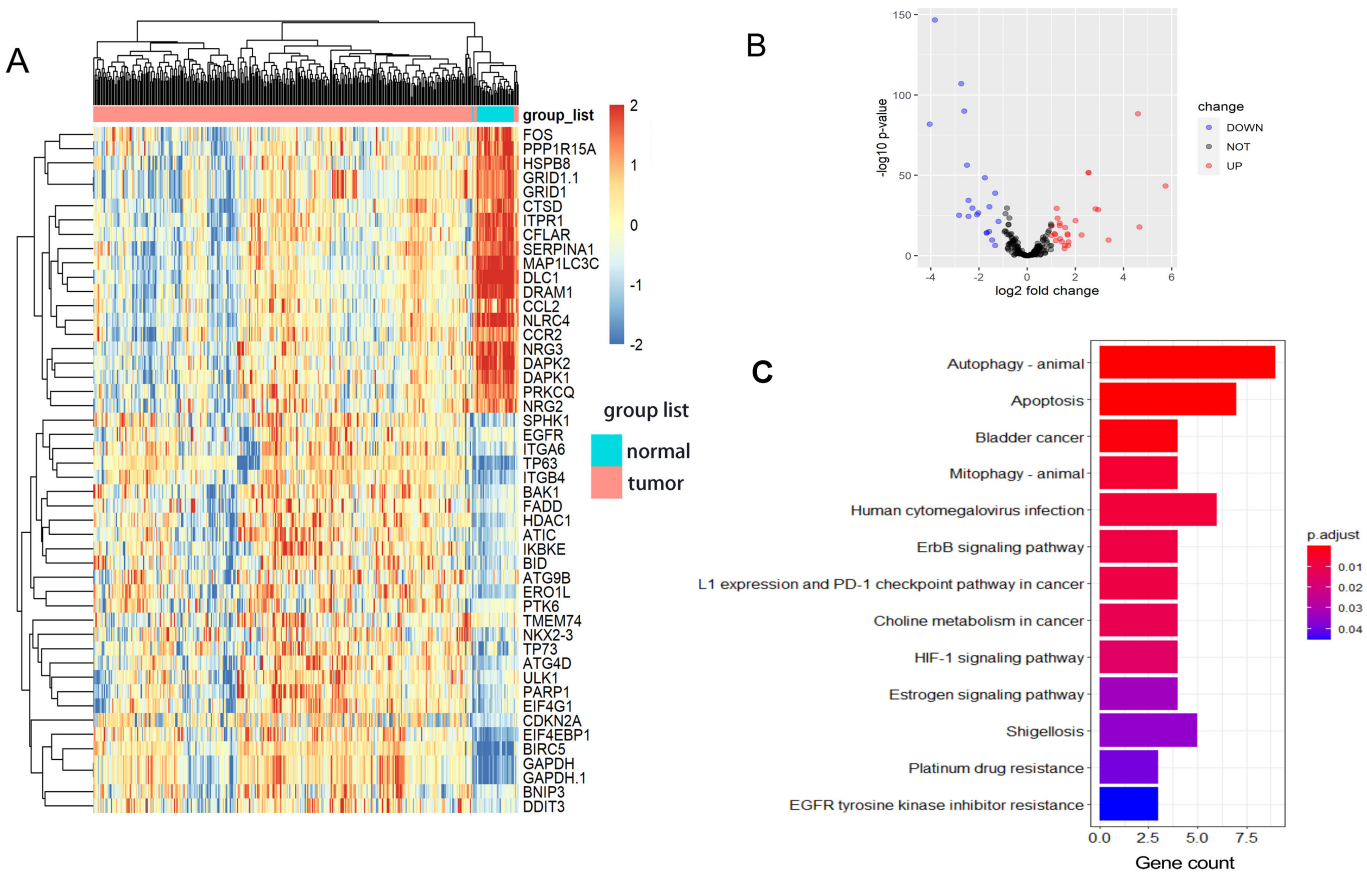
Results of differentially expressed analysis on ARGs and enrichment analysis of DE‐ARGs. (A) A heatmap of 48 differentially expressed ARGs between 326 LUSC samples and 32 normal controls. Each line represents a DE‐ARG and each row means a sample. The expression levels of genes are displayed with colors in each cell (red for high and blue for low). (B) The volcano plot of differentially expressed ARGs between LUSC samples and normal controls. (C) The enriched significant KEGG signal pathways of DE‐ARGs. The color represents the statistical significance of the term. The length indicates the counts of enriched genes.

Using the edgeR algorithm and screening criteria for DEGs, we identified 48 out of 224 ARGs (Table S1) with significant alterations in expression levels in LUSC compared to normal controls, including 28 up‐regulated and 20 down‐regulated genes, respectively (*p* < 0.05 and log2 |fold change| > 1). These results are presented in Figure 1A,B. Subsequently, these differentially expressed ARGs (DE‐ARGs) underwent KEGG pathway enrichment analysis, revealing enrichment primarily in the regulation of autophagy, apoptosis, and mitophagy (Figure 1C). Notably, enrichment of the “PD‐L1 expression and PD‐1 checkpoint pathway in cancer” suggests a correlation between these ARGs and immunosuppression pathways.

### 3.2 Construction of the Autophagy‐Related Prognostic Signature

The 326 LUSC samples from TCGA were divided into high and low gene expression groups according to the expression level of each ARG (cut‐off value was 50%). Then, the Kaplan–Meier method and log‐rank test were applied to examine overall survival (OS) between the two groups. All ARGs (rather than DE‐ARGs) were examined. As shown in Table S2, 54 genes were significantly related to OS of LUSC patients (*p* < 0.05), and these genes were defined as survival‐related ARGs (sARGs). Additionally, we observed that not all sARGs were DE‐ARGs. The intersection of sARGs and DE‐ARGs is presented in Figure S1. Immunohistochemistry data of ARGs in normal lung tissue and LUSC tumor tissue from The Human Protein Atlas project are also included (Figure S2). Furthermore, survival curves of LUSC patients divided into high and low expression of ARGs were presented in Figure S2 (Data from The Human Protein Atlas project, https://www.proteinatlas.org/).

**FIGURE 2:**
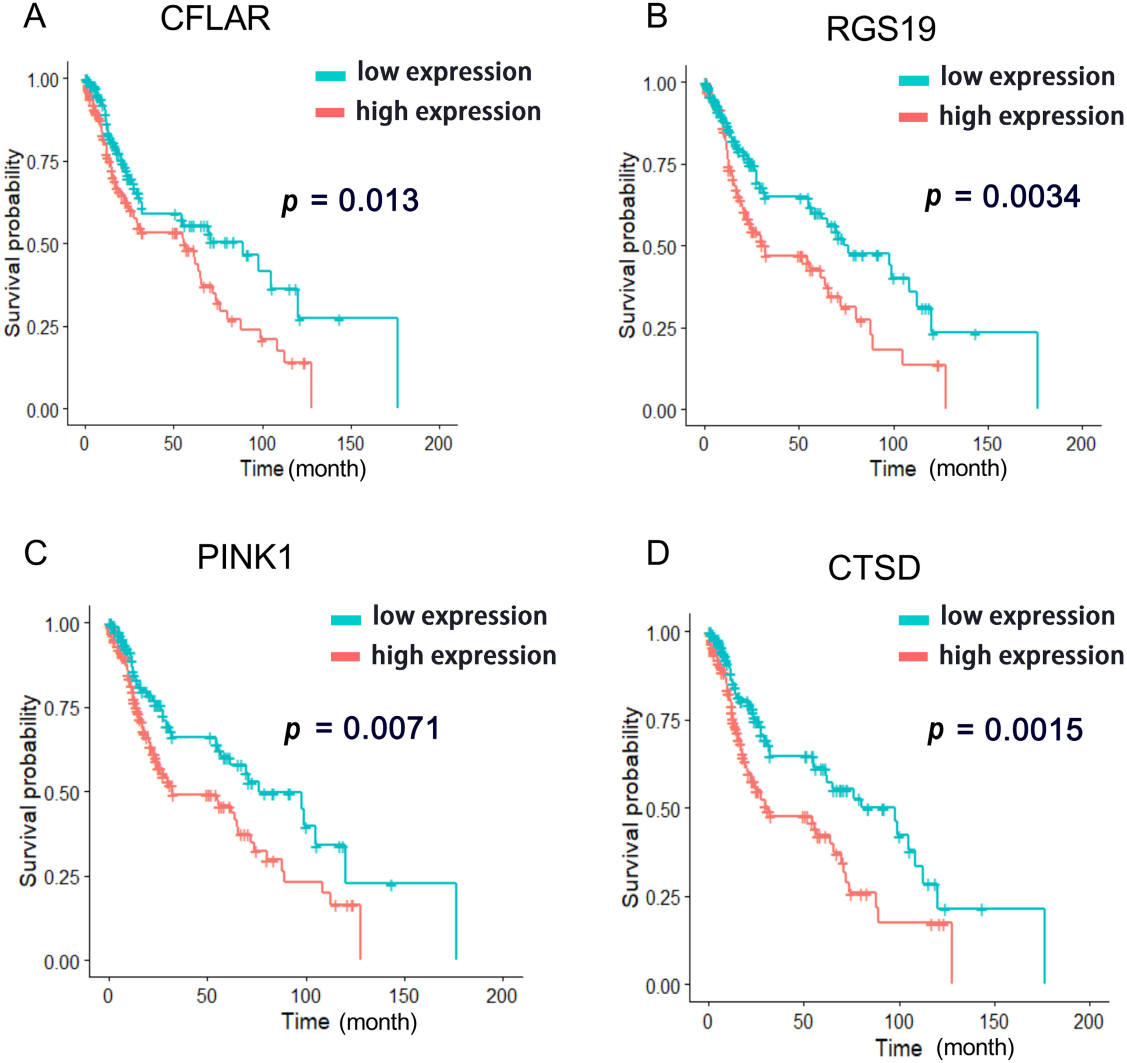
The results of survival analyses. (A–D) shows the Kaplan–Meier overall survival (OS) curves of CFLAR, RGS19, PINK1, CTSD for LUSC patients based on high and low expression levels of these four genes (cutoff = 50%).

**FIGURE 3:**
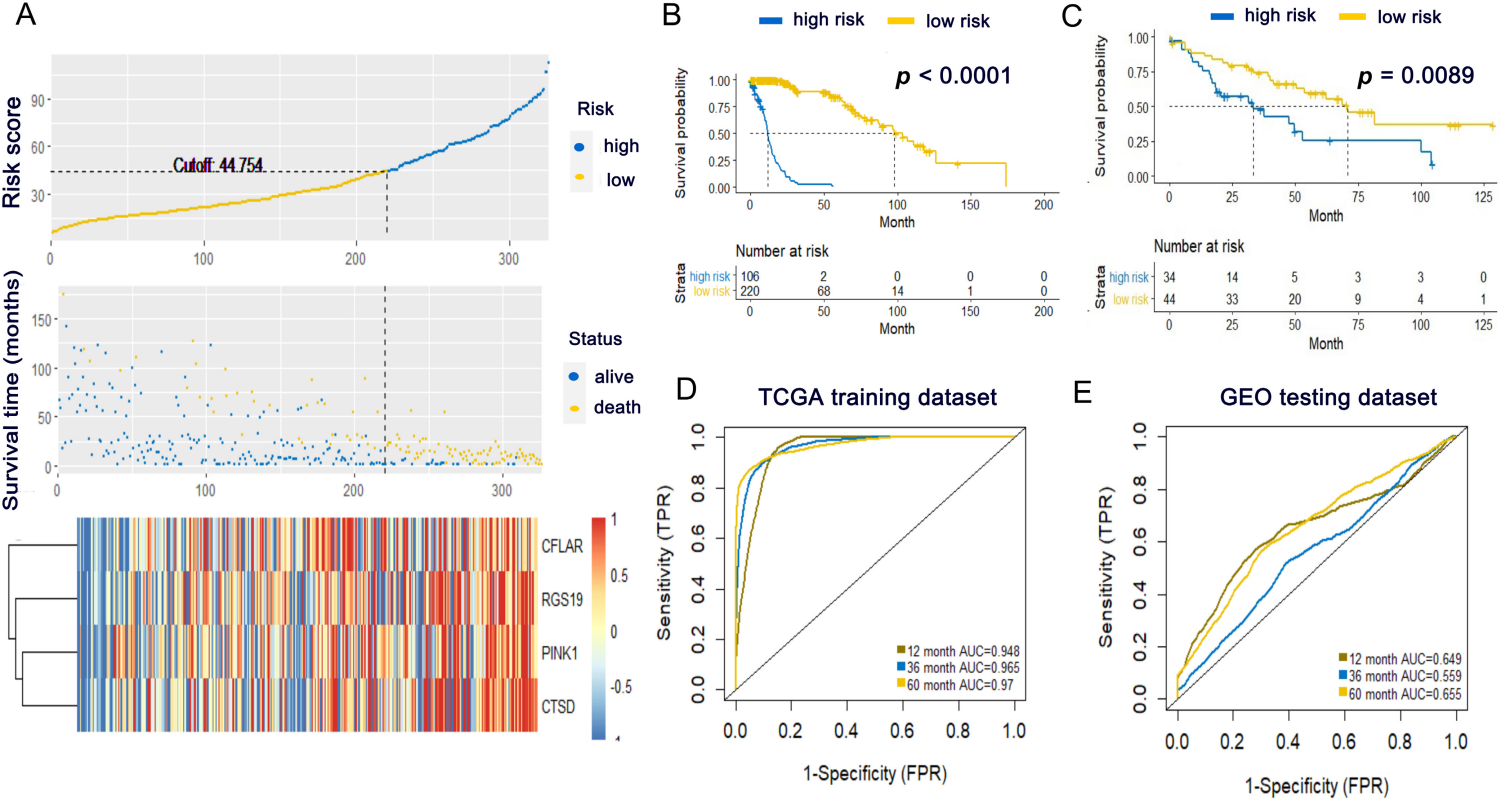
Construction and validation of the Autophagy‐related prognostic signature. (A) Risk score distribution, survival status of each patient, and heatmaps of prognostic four‐gene signature in TCGA cohorts. Patients were ranked by risk score. (B, C) Kaplan–Meier survival curve of OS among LUSC patients from low‐risk group and high‐risk group in TCGA training dataset (B) and GEO testing dataset (C). (D, E) Receiver operating characteristic (ROC) curves of the risk score model in TCGA training dataset (D) and GEO testing dataset (E).

Next, Random Forest was performed to select sARGs with the best prognostic value and to build an autophagy‐related risk score model in the TCGA cohort. Four sARGs, including CFLAR, RGS19, PINK1, and CTSD, were selected as the most important survival‐relevant variables. The Kaplan–Meier overall survival (OS) curves of these four genes were displayed in Figure 2 (The cutoff value of gene expression level was 50%). The random survival forest‐based score was derived for each sample and defined as the risk score. Patients in the TCGA cohort (training dataset) were then assigned to a high‐risk or low‐risk group using the optimal cut‐off value obtained with the survminer R package. Risk score distribution and corresponding four‐gene expression patterns are shown in Figure 3A. With the increase in risk score, the expression levels of the four genes were elevated. The overall survival time of the high‐risk group was shorter than that of the low‐risk group.

### 3.3 Validation of the Autophagy‐Related Prognostic Signature

For further validation, 78 samples of LUSC subtype from GSE41271 (GEO dataset) were used as an independent testing dataset. Then, Kaplan–Meier analysis was applied to the two cohorts and demonstrated that patients with a high‐risk score were correlated with worse outcomes in the two cohorts (Figure 3B,C). Additionally, time‐dependent ROC curve analysis of the risk score model in the TCGA cohort indicated promising prognostic ability for OS (1‐year AUC = 0.948, 3‐year AUC = 0.965, 5‐year AUC = 0.97, Figure 3D). Meanwhile, the ROC curve of OS prediction was drawn in the GEO cohort (1‐year AUC = 0.649, 3‐year AUC = 0.559, 5‐year AUC = 0.655, Figure 3E). The autophagy‐related model showed a less promising prognostic ability in the GEO cohort than the TCGA cohort, possibly due to the small sample size of the GEO dataset (78 in GEO vs. 326 in TCGA), which warrants further validation with larger samples. Furthermore, in the TCGA cohort, age, gender, and TNM stage (M stage excluded) were considered as candidate risk factors for Cox regression analyses. As shown in Table 1, univariate Cox regression analysis indicated that N stage and risk score are significant risk factors for poor prognosis, and multivariate Cox regression demonstrated the independence of the risk score of this signature in prognosis prediction from other clinical factors.

**TABLE 1:** Univariate Cox regression and multivariate Cox regression of risk score and clinical traits.

Variables	Univariate Cox mode		Multivariate Cox mode	
	HR (95% CI)	*p*	HR (95% CI)	*p*
Gender	0.864 (0.556–1.344)	0.516	1.3669 (0.8617–2.168)	0.184
Age	1.01 (0.988–1.032)	0.378	0.9946 (0.9715–1.018)	0.654
T stage	1.275 (0.999–1.629	0.051	1.0003 (0.7929–1.262)	0.998
N stage	1.331 (1.042–1.7)	0.022*	1.1868 (0.7597–1.854)	0.452
Risk score	1.107 (1.093–1.122)	< 0.0001****	1.1096 (1.0944–1.125)	< 0.0001****

* *p* < 0.05,

**** *p* < 0.0001.

### 3.4 High‐Risk Group Indicated an Immune‐Suppression Status

**FIGURE 4:**
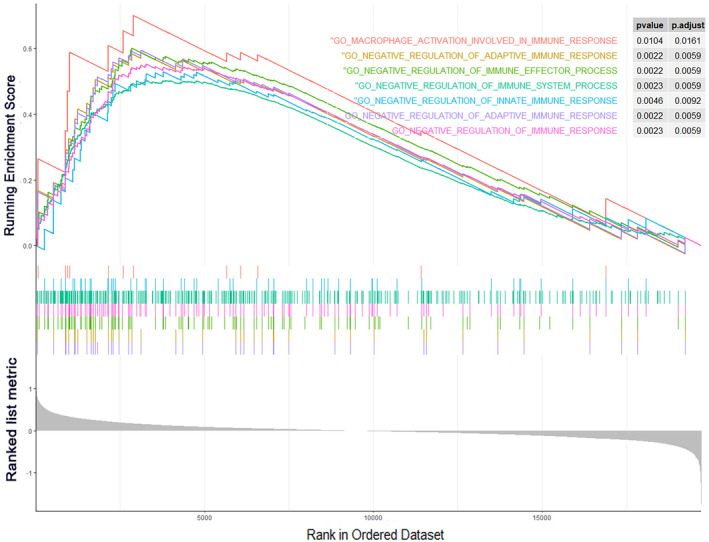
Pathways involved in negatively regulation of immune response by GSEA analyses. GSEA analyses displayed gene sets that were significantly enriched in high (up) or low (down) risk group. The pathways were colored, respectively (Orange: Macrophage activation involved in immune response; Yellow: Negative regulation of adaptive immune response; Light green: Negative regulation of immune effector process; Aquamarine blue: Negative regulation of immune system process; Blue: Negative regulation of innate immune response; Violet: Negative regulation of adaptive immune response; Red violet: Negative response of immune response).

To explore the immune phenotype and potential immune‐related mechanisms underlying our constructed prognostic gene signature, we performed Gene Set Enrichment Analysis (GSEA) to identify enriched immune‐related gene sets annotated by the gene ontology (GO) term. Results revealed that seven significantly altered immune‐related pathways were enriched in the high‐risk group, while no immune‐related pathways were enriched in the low‐risk group (Figure 4). A high‐risk score was significantly associated with macrophage activation involved in immune response (*p* < 0.05), negative regulation of adaptive immune response (*p* < 0.01), negative regulation of immune effector process (*p* < 0.01), negative regulation of immune system process (*p* < 0.01), negative regulation of innate immune response (*p* < 0.01), negative regulation of adaptive immune response (*p* < 0.01), and negative response of immune response (*p* < 0.01). Consistently, the high‐risk group exhibited an immunosuppressive phenotype.

### 3.5 High‐Risk Group Showed an Increased Infiltration of Suppressive Immune Cell Populations

**FIGURE 5:**
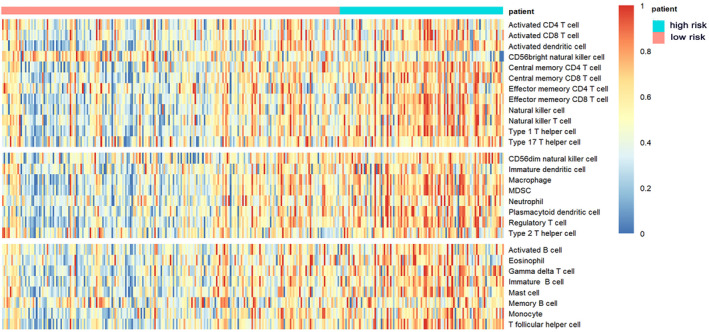
Heatmap of immune cell infiltration level of LUSC tumor samples in TCGA cohort. A Single‐Sample Gene Set Enrichment Analysis identifying the relative infiltration of 28 immune cell populations for LUSC tumor samples. Samples in the heatmap were ranked by risk score of each patient. The ssGSEA score which represents the relative infiltration of each cell type was normalized to unity distribution, for which zero is the minimal and one is the maximal score for each immune cell type (red represents high and blue represents low infiltration). The three parts of the heatmap exhibited the three types of immune cells (anti‐tumor immunity, pro‐tumor immune‐suppression, and other unclassified immune cells).

To further investigate the association between the autophagy‐related score and the regulation of suppressive immune cell populations, we calculated the normalized Single‐Sample Gene Set Enrichment Analysis (ssGSEA) scores of 28 infiltrating immune cell populations in each sample and represented their infiltration level. We visualized the relative abundance of the 28 infiltrating immune cell populations in a heatmap (Figure 5). The boxplot of these immune cells labeled with the *p* value of the Wilcoxon rank test between the low‐risk and high‐risk score groups was shown in Figure S3.

**FIGURE 6:**
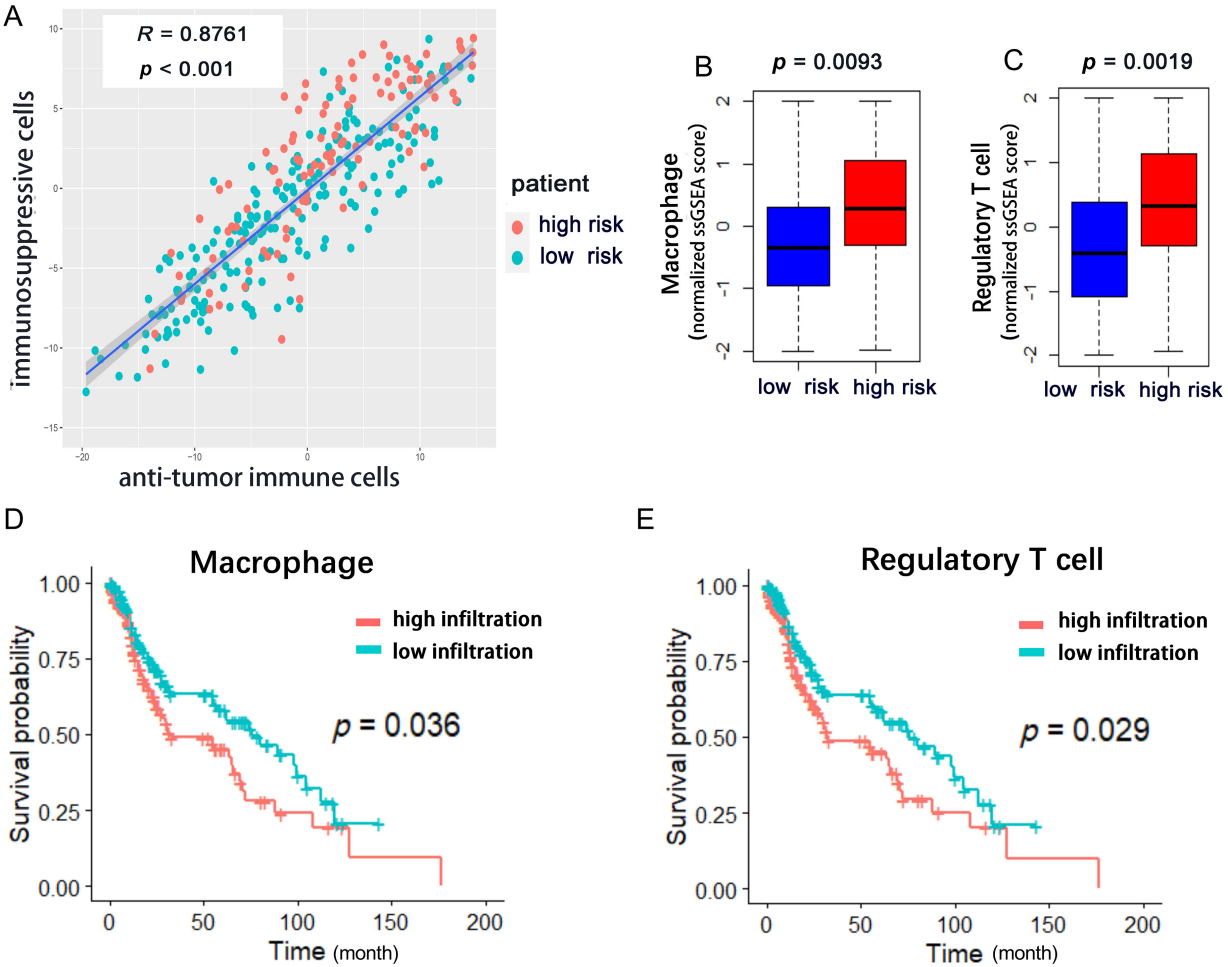
The analyses of immune cell infiltration. (A) Correlation between infiltration of cell types executing anti‐tumor immunity and pro‐tumor, immune suppressive functions. R coefficient of Pearson's correlation and *p* value were shown. (B) High‐risk group was associated with higher infiltration of Macrophage (C, *p* < 0.01). (C) High‐risk group was associated with higher infiltration of Regulatory T cell (Wilcox‐test, *p* < 0.01). (D) Macrophage infiltration was negatively correlated with overall survival (Log‐Rank test, *p* < 0.05). (E) Regulatory T cell infiltration was negatively correlated with overall survival (Log‐Rank test, *p* < 0.05).

We observed that the high‐risk score group had a relatively higher infiltration of anti‐tumor immune cells (Activated CD4 T cell, Activated CD8 T cell, Central memory CD8 T cell, Effector memory CD4 T cell, Effector memory CD8 T cell, Type 1 T helper cell, Type 17 T helper cell, CD56bright natural killer cell). The high‐risk score group also had higher infiltration of immunosuppressive cells (Regulatory T cell, Type 2 T helper cell, CD56dim natural killer cell, Immature dendritic cell, Macrophage, MDSC, Neutrophil, Plasmacytoid dendritic cell), which execute pro‐tumor and immune suppressive functions. For further investigation, Pearson's correlation analysis was applied to the abundances of these two categories of cells. Results showed that the abundances of anti‐tumor immune cells were positively associated with the abundances of immunosuppressive cells in the tumor microenvironment (Figure 6A). It appeared that immune suppression is stronger in the high‐risk group than the low‐risk group, which is consistent with GSEA results.

### 3.6 Macrophage and Regulatory T Cell (Treg) Correlated With Worse Outcome

Then, we conducted multivariate Cox regression, univariate Cox regression, and Kaplan–Meier analysis plus the log‐rank test to select the prognostic immune cells among these 28 infiltrating immune cell subtypes. The forest plot of multivariate Cox regression was presented in Figure S4. Results revealed that only macrophage and regulatory T cell had a significant correlation with overall survival. As shown in Figure 6D,E, higher infiltration of macrophage and regulatory T cell correlated with shorter overall survival time (*p* < 0.05). As mentioned above, the high‐risk group was associated with higher infiltration of macrophage and regulatory T cell (*p* < 0.01) (Figure 6B,C). This observation suggested that macrophage and regulatory T cell may account for the poor prognosis induced by immunosuppression in the high‐risk group.

### 3.7 Correlation Between Genes Expression and Immunocyte Infiltration

**FIGURE 7:**
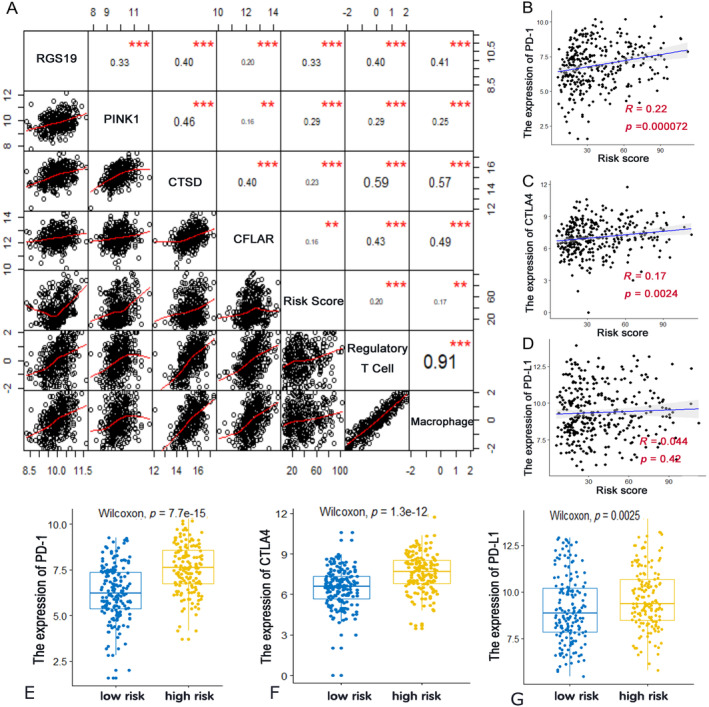
Correlations of risk score with genes expression, immunocyte infiltration and the immune checkpoint. (A) Correlation of the risk score with genes expression and immunocyte infiltration level. Pearson's correlation coefficient values with the significance level were shown on the top of the diagonal (**p < 0.01, ***p < 0.001). (B–D) Correlation of the risk score with the expression of several key immune checkpoints. (B) PD‐1; (C) CTLA4; (D) PD‐L1. Pearson's correlation coefficient values with the *p* value were shown. (E–G) The boxplots showed the comparison of the expression of several key immune checkpoints between the high‐risk and low‐risk group. (E) PD‐1; (F) CTLA4; (G) PD‐L1. Wilcox‐test was conducted and *p* values were provided in each figure.

We investigated the correlations of the expression of the four genes in this autophagy‐related signature with the infiltration levels of macrophages and regulatory T cells, as well as the risk score. Figure 7A demonstrates the overall correlations among these variables. The four genes were strongly interrelated and exhibited a significantly positive association with the infiltration of the two immunocytes. Additionally, gene expression and immunocyte infiltration increased along with the growth of the risk score.

### 3.8 Immune Checkpoints Analysis

Immune checkpoint inhibitors are emerging as promising strategies in the treatment of lung cancer. Therefore, we investigated the association of the risk score with the main immune checkpoints, including PD‐1/PD‐L1 and CTLA4. As shown in Figure 7B–D, the risk score showed a significantly positive correlation with PD‐1 and CTLA4 (*p* < 0.01). However, there was no significant correlation between the risk score and PD‐L1 expression. We conducted further statistical comparisons of their expression between the high‐risk and low‐risk groups. As shown in Figure 7E–G, the expression of PD‐1, PD‐L1, and CTLA4 was higher in the high‐risk group. We also examined the correlations between the expression of the four ARGs (CFLAR, RGS19, PINK1, CTSD) and the expression of immune checkpoints (PD‐1, PD‐L1, CTLA4). Results showed that the expressions of the four ARGs had significant associations with the immune checkpoints consistently (only the correlation between PINK1 and PD‐L1 was not significant) (Figure S5).

### 3.9 Estimate Score, Immune Score and Stromal Score

**FIGURE 8:**
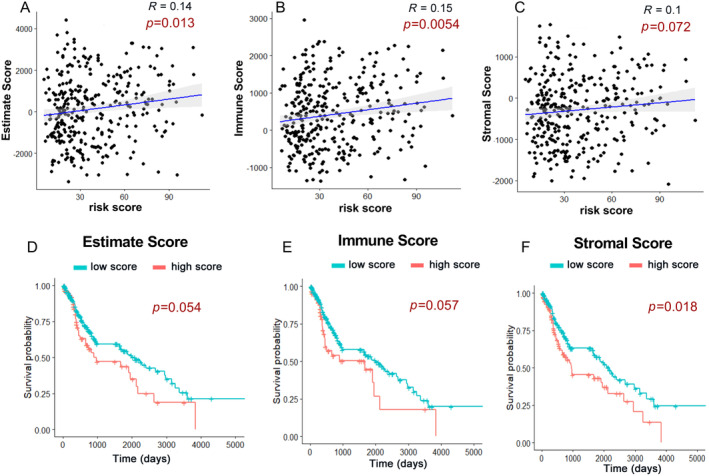
Correlation and survival analyses of estimate score, immune score and stromal score. (A–C) The correlations of risk score with estimate score, immune score, and stromal score. Pearson's correlation coefficient values were showed. (D–F) The Kaplan–Meier overall survival curves for LUSC patients assigned to high and low score group. (D) Estimate score; (E) Immune score; (F) Stromal score.

ESTIMATE (Estimation of Stromal and Immune cells in Malignant Tumor tissues using Expression data) is a newly developed algorithm that uses the transcriptional profiles of cancer tissues to infer the level of infiltrating stromal and immune cells based on specific gene expression signatures of stromal and immune cells in the specific cancer type. In the present study, the stromal score, immune score, and estimate score of each included sample of was calculated by applying Estimate R package. Next, the association of autophagy‐related risk scores with the estimate/immune/stromal scores was examined. As shown in Figure 8A–C, estimate score and immune score were found to have a positive correlation with the risk scores of our established model (*p* < 0.05). Stromal score was also rising with the increase of risk score, but not significantly (*p* = 0.072). The prognostic value of the three types of scores was also investigated. Survival analysis showed that the patients with higher estimate/immune/stromal score had a poorer overall survival, although the statistical significance was only observed in stromal score (*p* < 0.05).

## 4 Discussion

Over the past decade, new treatments for patients with LUSC have evolved dramatically, including immune checkpoint inhibitors and combination strategies. Thus, the more refined risk‐stratification to guide treatment for LUSC patients beyond conventional TNM staging are needed. With the rapid development of high‐throughput next generation sequencing, machine learning, and bioinformatics analyses, prognostic signatures composed of a set of genes have been designed to meet this need. Compared to single molecular predictors, signatures integrated the prognostic value of several genes and seem to have better outcome prediction ability. In previous studies, several risk signatures have shown satisfactory effects in outcome prediction and treatment guiding in lung cancer, such as miRNA‐ based signature [23, 24], lncRNA‐related signature [24], immune‐associated signature [25]. These risk signatures could be used as the tailored algorithms to individualize therapy.

Autophagy, which induces degradation of proteins and organelles or cell death upon cellular stress, is crucial in the pathophysiology of malignant tumor [10, 26]. The apoptotic effect of autophagy is controversial as both inhibitory and stimulatory effects have been reported in NSCLC [26, 27]. Consequently, an integrated study of a multi‐molecule genes model is needed to figure out the exact effects of autophagy in lung cancer. A previous study has reported an autophagy‐associated multiple‐gene signature that correlated with survival in NSCLC [27]. However, in the era of immunotherapy, the immune contexture of tumor microenvironment is essential for the treatment selection and survival prediction [28]. The correlations between autophagy and immune pathway should not be ignored [11]. Thus, in this study, we conducted a comprehensive and meaningful analysis of autophagy‐related genes involved in outcome prediction and associated immune landscape, which may also enable the identification of patients who are more likely to respond to immunotherapy. Moreover, different with previous studies, random forest instead of Lasso regression was applied in the current analyses. Random forest is a machine learning process with accurate algorithms and high efficiency in classification prediction.

In the present study, four ARGs including CFLAR, RGS19, PINK1, and CTSD were screened out as the most important survival relevant variables and were selected for the construction of an autophagy‐related prognostic risk signature. The four sARGs are not all DE‐ARGs. Only CFLAR and CTSD are DE‐ARGs. Interestingly, CFLAR and CTSD were downregulated in LUSC compared to normal tissue. Our results were similar to the immunohistochemistry data form the Human Protein Atlas project (Figure S2). We speculated that it may be explained by the fact that autophagy is a double‐edged sword towards tumor cell. Autophagy is supposed to protect cells and tissues from stressors in normal physiological processes, while tumor genesis happens when these genes are downregulated. However, during the advanced stages of tumors, autophagy is upregulated and promotes tumor cell proliferation. This was the reason why we used all ARGs (rather than DE‐ARGs) for the survival analysis.

CFLAR, also known as c‐FLIPL, is a critical anti‐apoptotic protein that inhibits cell death mediated by the death receptors Fas, DR4, DR5, and TNF‐R1 [29, 30]. CFLAR was found as an independent adverse prognostic biomarker in different cancer types [31, 32]. Besides, elevated expression of CFLAR is associated with tumor cells escaping from immune surveillance in vivo, correlates with a more aggressive tumor, and is also considered to be the main cause of immune escape [30]. A recent study reported that down regulation of CFLAR could enhance the antitumor response of T cells and enhances the PD‐1 blockade efficacy in a melanoma tumor model [33]. And knockdown of CFLAR could also decrease the expression of PD‐L1 [33]. Consistent with these results, our analyses found that high expression of CFLAR was significantly associated with some key immune checkpoint including PD‐1, PD‐L1, and CTLA4 (Figure S5). These findings may provide a new combined therapeutic target for further improving the efficacy of ICIs. RGS19 is a member of the regulators of G protein signaling (RGS proteins) [34]. Rapid termination of G protein signals by RGS proteins can potentially modulate growth signals and hence promote tumorigenesis, although no clear mechanism of RGS19 in lung cancer was reported [34]. Besides, physiological functions of most RGS proteins in immune response are largely unknown [35]. Our study first demonstrated RGS19 could be a risk factor for poor prognosis, and also correlated with the expression of several immune checkpoint and the infiltration of Tregs and Macrophages (Figures S5 and 7A). This discovery provides a new perspective that RGS19 may participate in immune process regulation, which worth further studies. PINK1 (PTEN induced kinase 1) mediates the recruitment of Parkin to mitochondria, which facilitates the elimination of the injured mitochondria by autophagy. It also has been proved to play crucial roles in the genesis and development of tumor [36]. In lung carcinoma tissue, the expression of PINK1 was raised, which was associated with a poor prognosis [37]. In recent years, PINK1 was also found as a repressor of the immune system and played a key role in adaptive immunity by repressing presentation of mitochondrial antigens [38]. Similar with previous studies, our study reported PINK1 was a risk factor for worse outcome. Moreover, PINK1 was found to be associated with the expression of PD‐1 and CTLA4 as well as the infiltration of Tregs and macrophages. CTSD (Cathepsin D) is a key protein for lysosomal function that is necessary for autophagy in cancer cells [39]. Although CTSD is expressed at high levels in many cells of the immune system, but its role in immune function is still not well understood. In the present investigation, we observed that, similar with the other three genes, CTSD was correlated with poor survival and immunosuppressive status as well.

Taken together, it is reasonable to believe that the combination of these four genes has robust prediction value in LUSC, and the four‐gene signature could stratify patients with different immune contextures, which is critical in the context of current immunotherapy. Moreover, GSEA analysis was employed to make the functional annotation, and we found the more abundant negative‐regulation of immune responses and processes in the high autophagy‐related score group. Later, survival analyses were employed to examine whether the infiltration of 28 infiltrating immune cell subtypes were prognostic and it turned out that only macrophage and Treg cells have a significantly negative correlation with overall survival. While, these two types of cells delivering pro‐tumor suppression were consistently enriched in high‐risk group. Recent studies have shown that autophagy significantly controls immune responses by modulating the functions of immune cells and the relationship between autophagy and immunity are complicated [40]. On one hand, autophagy has been shown to be important for priming of tumor‐specific CD8+ T cells, and inhibition of autophagy would impair systemic immunity [41]. However, on the other hand, the induction of autophagy may also benefit tumor cells escape from immune surveillance [40]. A recent report indicated that immunosuppressive Treg cells are critically dependent on autophagy [42]. Results showed that autophagy is active in Treg cells and supports their lineage stability and survival fitness [42]. While, for macrophage, autophagy regulates cellular development of monocytes, resulting in the disturbance of macrophage differentiation. Thus, our findings that high‐risk group with higher expression of the four autophagy‐related genes had an increased infiltration level of Treg cells and macrophages should be noticed. Moreover, based on estimate/immune/stromal scores from the ESTIMATE algorithm, we found that both the estimate score and immune score of high‐risk group are higher and patients with higher scores tended to have a worse outcome.

The high‐risk group presented a comparatively suppressed immune status, the phenotype of adaptive immune evasion. Actually, the infiltration of almost all immune cells were upregulated in high‐risk group, including both the immune‐suppressive subtypes and immune‐stimulatory subtypes. This observation possibly suggested the presence of a feedback mechanism such that the recruitment or differentiation of cells specialized for immune suppression may be facilitated by anti‐tumor inflammation. This could also be explained by our results that the immunosuppressive molecules points such as PD‐1, CTLA4 was also higher in high‐risk group, which means the presence of a suppressed pre‐existing antitumor immunity that could be re‐invigorated by anti PD‐1/PD‐L1 immunotherapy [43]. Besides, a previous review has been proposed that four different types of tumor microenvironment exist based on the presence or absence of tumor‐infiltrating lymphocytes (TILs) and PD‐L1 expression [44]. Type I cancers (with higher PD‐L1+ and TILs) are more likely to benefit to anti‐PD‐1/L1 therapy [44]. Thus, we speculated that the high‐risk group are more likely to respond to ICIs.

This study has certain limitations. This research relies solely on bioinformatics analysis without further experimental validation or functional verification. Additionally, there is a lack of investigation into the correlation between non‐coding RNAs (microRNAs or long non‐coding RNAs) and inflammation or autophagy [45, 46]. In future studies, we plan to collect tissue samples from LUSC patients undergoing immunotherapy for validation testing of our predictive model and further explore the roles of these autophagy‐related genes in ICIs treatment. Additionally, we aim to investigate the potential of repurposing drugs as prophylactics for cancer management [47].

In conclusion, based on bioinformatics analysis, this study developed a new autophagy‐related four‐gene prognostic model signature, which could be applied as an independent prognostic indicator for LUSC patients. In addition, the autophagy‐related scores are bound up with immune phenotype of LUSC, with higher score indicating an immune‐suppression status. This study provides a novel and comprehensive sight to the correlation of autophagy and immune landscape in the tumor microenvironment of LUSC.

## Author Contributions

Lumeng Luo and Jiaying Deng contributed to the design of the study. Lumeng Luo and Jiaying Deng contributed to the data collection and analysis. Lumeng Luo contributed to the paper writing. Qiu Tang contributed to the revising work and the manuscript review. All authors read and approved the final manuscript.

## Ethics Statement

The authors have nothing to report.

## Consent

The authors have nothing to report.

## Conflicts of Interest

The authors declare no conflicts of interest.

## Supporting information


**Figure S1.** (A) The table showed the list of DE‐ARGs which differently expressed between normal tissue and LUSC and the list of survival‐related ARGs. (B) The Venn diagram showed the intersection of DE‐ARGs and survival‐related ARGs.

**Figure S2.** The immunohistochemistry data of four ARGS (CFLAR, RGS19, PINK1, and CTSD) in normal lung tissue and LUSC tumor tissue form the Human Protein Atlas project (https://www.proteinatlas.org/) (A, C, E, G). Besides, B, D, F, H showed the survival curves of the LUSC patients which was divided into high and low expression of ARGs. The high expressions of CTSD/CFLAR/RGS19/PINK1 were correlated with the worse prognosis in LUSC patients (Data from The Human Protein Atlas project).

**Figure S3.** Tumor microenvironment (TME) composition group by risk score. The infiltration level of 28 immune cells in high‐risk group versus low‐risk group (Wilcoxon‐test, ns: no significance, *p < 0.05, **p < 0.01, and ***p < 0.001, ****p < 0.0001).

**Figure S4.** Multivariate Cox regression was conducted among these 28 infiltrating immune cell subtypes to select the prognostic immune cells. The forest plot showed the results of multivariate Cox regression (*p < 0.05).

**Figure S5.** Correlations between the expression of the ARGs (CFLAR, RGS19, PINK1, CTSD) and the expression of immune checkpoints (PD‐1, PD‐L1, CTLA4). Pearson’s correlation coefficient values with the *p* value were shown.

**Table S1.** The gene list of 224 ARGs.

**Table S2.** The gene list of 54 survival‐related ARGs (sARGs).

## Data Availability

Publicly available datasets were analyzed in this study. These can be found here: TCGA database (https://portal.gdc.cancer.gov/) and the NCBI Gene Expression Omnibus (https://www.ncbi.nlm.nih.gov/geo/).
