# Supplementary figures and images for: A Four‐Gene Autophagy‐Related Prognostic Model Signature and Its Association With Immune Phenotype in Lung Squamous Cell Carcinoma
Source: Cancer Rep (Hoboken). 2024 Oct 23;7(10):e70000. doi: 10.1002/cnr2.70000 (PMC11499073; doi:10.1002/cnr2.70000)

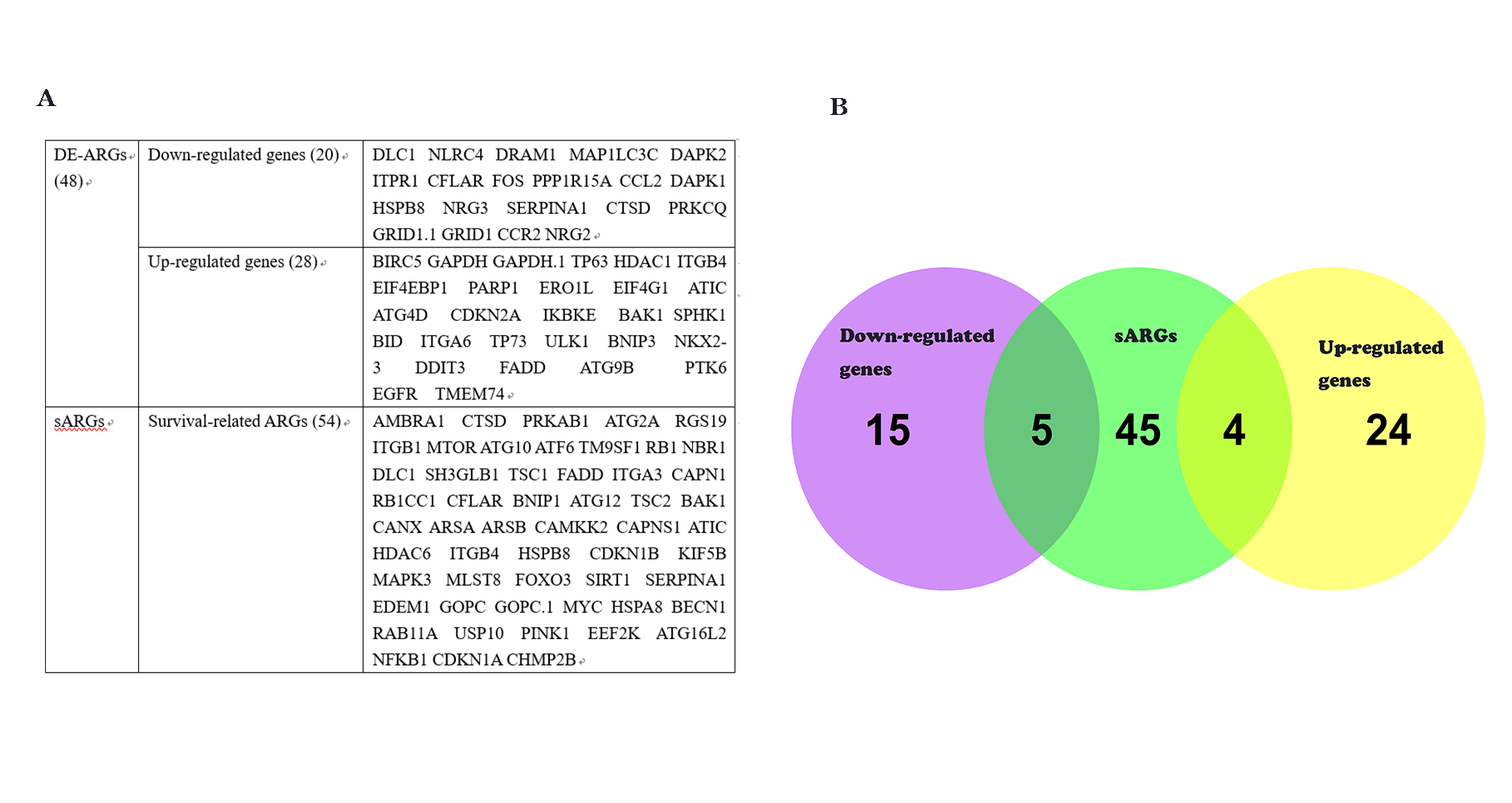

Supplement: Supplementary file 1 — Figure S1. (A) The table showed the list of DE‐ARGs which differently expressed between normal tissue and LUSC and the list of survival‐related ARGs. (B) The Venn diagram showed the intersection of DE‐ARGs and survival‐related ARGs. [file CNR2-7-e70000-s004.tif]

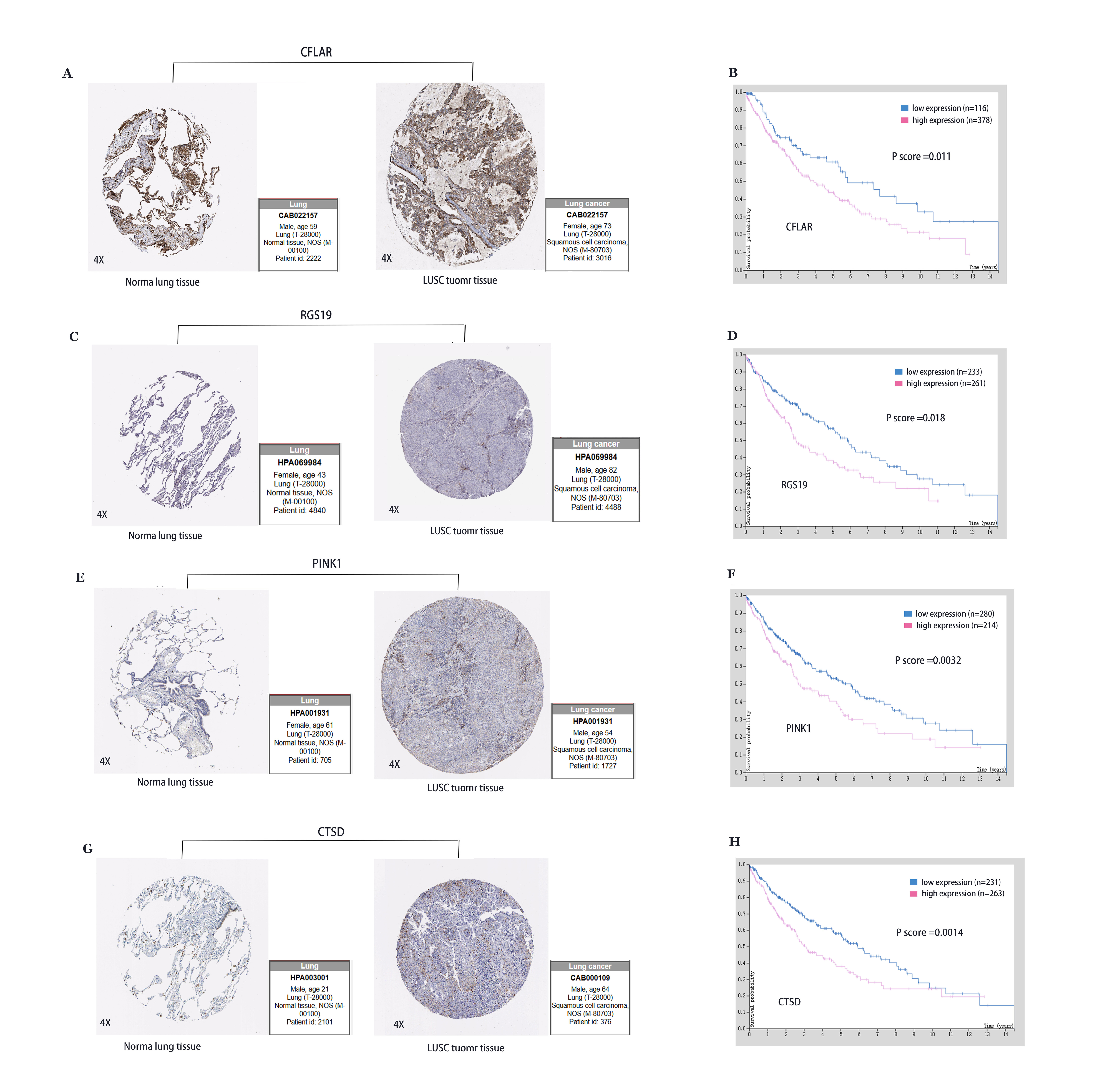

Supplement: Supplementary file 2 — Figure S2. The immunohistochemistry data of four ARGS (CFLAR, RGS19, PINK1, and CTSD) in normal lung tissue and LUSC tumor tissue form the Human Protein Atlas project (https://www.proteinatlas.org/) (A, C, E, G). Besides, B, D, F, H showed the survival curves of the LUSC patients which was divided into high and low expression of ARGs. The high expressions of CTSD/CFLAR/RGS19/PINK1 were correlated with the worse prognosis in LUSC patients (Data from The Human Protein Atlas project). [file CNR2-7-e70000-s002.jpg]

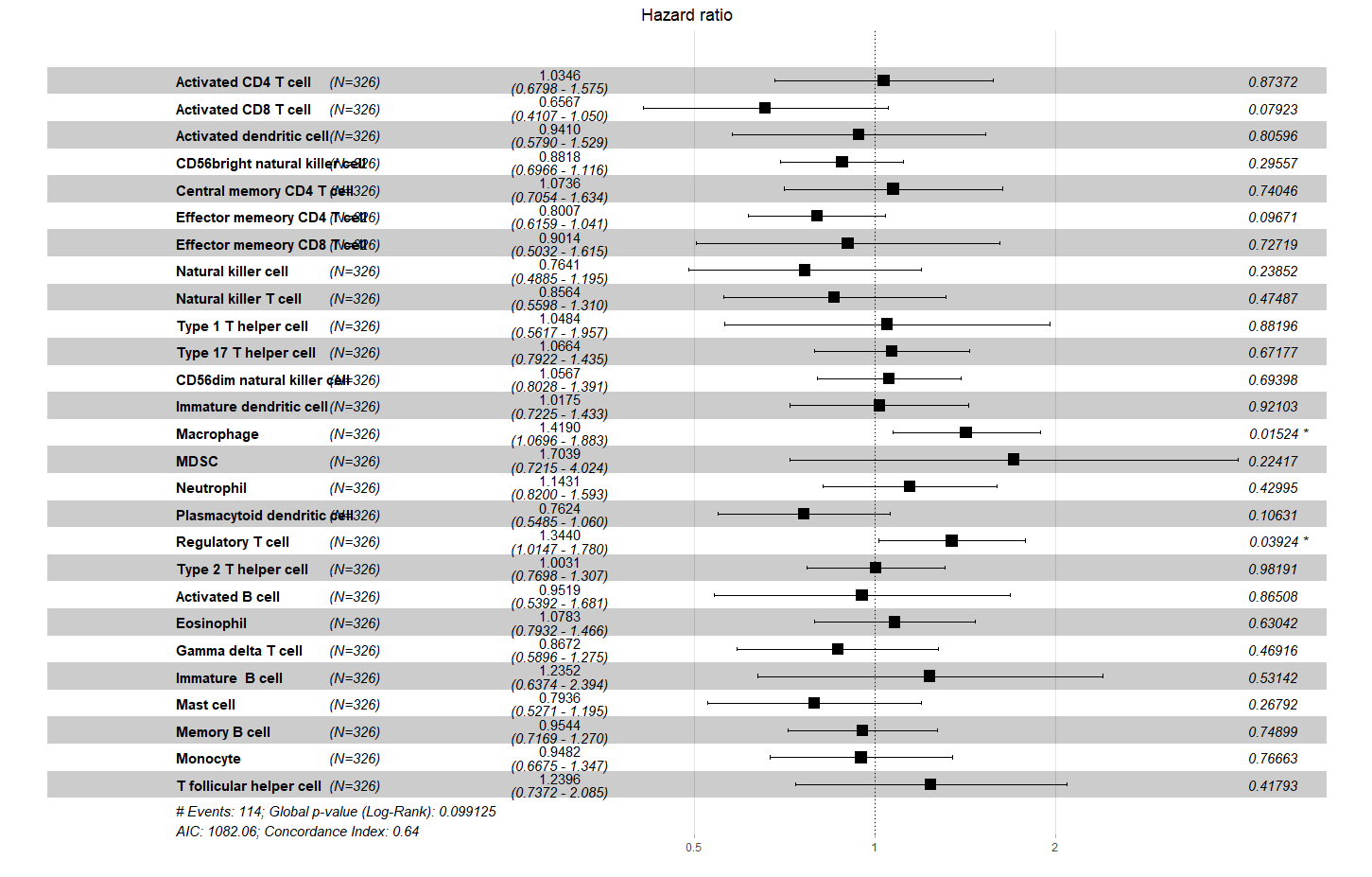

Supplement: Supplementary file 4 — Figure S4. Multivariate Cox regression was conducted among these 28 infiltrating immune cell subtypes to select the prognostic immune cells. The forest plot showed the results of multivariate Cox regression (*p < 0.05). [file CNR2-7-e70000-s003.tiff]

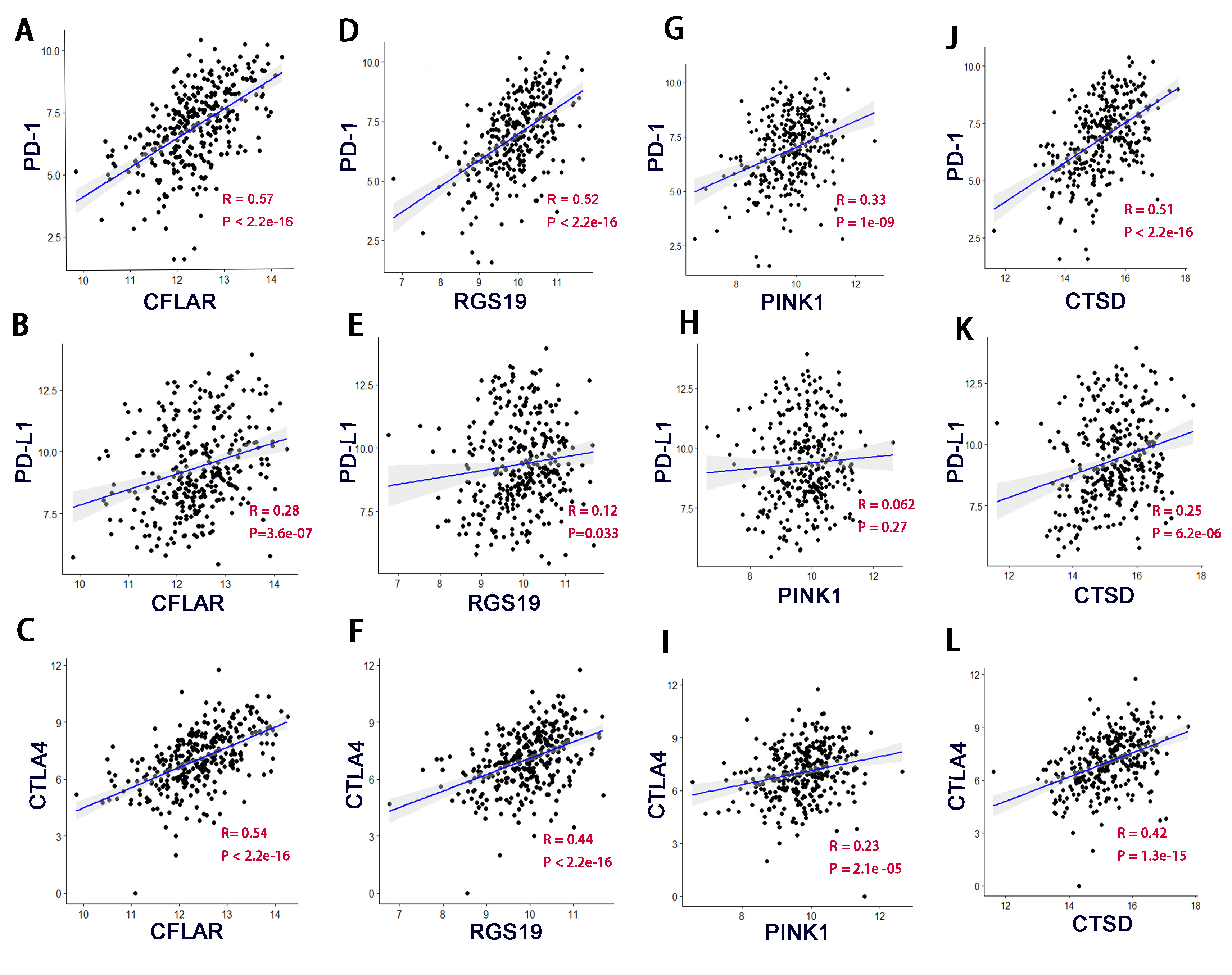

Supplement: Supplementary file 5 — Figure S5. Correlations between the expression of the ARGs (CFLAR, RGS19, PINK1, CTSD) and the expression of immune checkpoints (PD‐1, PD‐L1, CTLA4). Pearson’s correlation coefficient values with the p value were shown. [file CNR2-7-e70000-s005.jpg]
